# COVID-19 and Methylene Blue

**DOI:** 10.21470/1678-9741-2020-0462

**Published:** 2021

**Authors:** Viroj Wiwanitkit

**Affiliations:** 1 Padmashree Dr. DY Patil University, Dr. DY Patil University, Navi Mumbai, India E-mail: wviroj@yahoo.com

Dear Editor, I would like to comment on the article "COVID-19 should be a methylene blue "promoter"^[1]^". Evora noted that "*I decided to resubmit the letter now, considering that COVID-19 should be a methylene blue 'promoter', and the dye can get the lifesaving status it deserves* ^[1]^." In fact, the use of classic available agent as a possible therapeutic agent for the emerging COVID-19 should be supported. It is better than do nothing. Many classic drugs such as hydroxychloroquine are proposed and tested for managing COVID-19^[2]^. At the moment, we have to take the risk of a new alternative proposal for the treatment of COVID-19 such as convalescent plasma therapy^[3]^. Using a safe and well-known agent might not have a confirmation for its efficacy but we have many data on its safety, and it might be a good alternative option compared to a totally unknown new alternative.
